# Quality Improvement of Sample Collection Increases the Diagnostic Accuracy of Quantitative Fecal Immunochemical Test in Colorectal Cancer Screening: A Pilot Study

**DOI:** 10.3389/fmed.2021.762560

**Published:** 2021-10-26

**Authors:** Ru-chen Zhou, Pei-zhu Wang, Yue-yue Li, Yan Zhang, Ming-jun Ma, Fan-yi Meng, Chao Liu, Xiao-yun Yang, Ming Lv, Xiu-li Zuo, Yan-qing Li

**Affiliations:** ^1^Department of Gastroenterology, Qilu Hospital, Shandong University, Jinan, China; ^2^Laboratory of Translational Gastroenterology, Qilu Hospital, Shandong University, Jinan, China; ^3^Robot Engineering Laboratory for Precise Diagnosis and Therapy of GI Tumor, Qilu Hospital, Shandong University, Jinan, China; ^4^Clinical Epidemiology Unit, Qilu Hospital, Shandong University, Jinan, China

**Keywords:** colorectal cancer, occult blood, colonoscopy, endoscopy, stool testing

## Abstract

**Objective:** The diagnostic efficiency of the quantitative fecal immunochemical test (qFIT) has large variations in colorectal cancer (CRC) screening. We aimed to explore whether the practical sample collection operant training could improve the diagnostic accuracy of the qFIT in CRC screening.

**Methods:** Moderate-/high-risk individuals aged 50–75 years old were invited to participate in a prospective observational study between July 2020 and March 2021. Participants took a qFIT sample without fecal sample collection operant training in advance and then completed another qFIT sample after the operant training. The primary outcome was the sensitivity and specificity of the qFITs for CRC and advanced colorectal neoplasia (ACRN). The secondary outcome was the difference in the area under the curves (AUCs) and the concentrations of the fecal hemoglobin (Hb) between the qFIT without and after the operant training.

**Results:** Out of 913 patients, 81 (8.9%) patients had ACRN, including 25 (2.7%) patients with CRC. For CRC, the sensitivities of the qFIT without and after the operant training at 10 μg/g were 80.4 and 100.0%, respectively, and the specificities were 90.1 and 88.4%, respectively. For ACRN, the sensitivities were 49.4 and 69.1% and the specificities were 91.7 and 91.3%, respectively. The AUC of the qFIT after the operant training was significantly higher than that without the operant training for CRC (*p* = 0.027) and ACRN (*p* = 0.001). After the operant training, the concentration of the fecal Hb was significantly higher than that without the operant training (*p* = 0.009) for ACRN, but there was no significant difference for CRC (*p* = 0.367).

**Conclusion:** Practical sample collection operant training improves the diagnostic accuracy of the qFIT, which increases the detection of the low concentrations of fecal Hb. Improving the quality of the sample collection could contribute to the diagnostic efficiency of the qFIT in CRC screening.

## Introduction

Colorectal cancer (CRC) is the third most common cancer worldwide (1) and the second most lethal cancer among the types of cancer (2). Early detection is a well-recognized game-changer for the effective prevention and treatment of CRC (3–5). Although colonoscopy is the golden standard for CRC, it is not applicable for screening a large population. The fecal immunochemical test (FIT), as a non-invasive and cost-effective measure, is widely used for CRC screening (6). The role of the FIT in identifying early CRC and lowering CRC-associated mortality has been validated in large cohorts (7–9). The quantitative fecal immunochemical test (qFIT) is a laboratory-based testing method that automatically measures the concentration of the human hemoglobin (Hb) in the feces. In comparison to the qualitative FIT, qFIT can provide more information by selecting the optimal cutoff value to determine follow-up endoscopy (10–12).

The qFIT can detect the fecal Hb in a stable and sensitive manner (13, 14). However, large variations in the performance characteristics of qFIT in CRC screening have been observed. In the average risk for adults at the same fecal Hb threshold, the sensitivities of qFIT ranged widely from 75 to 100% for CRC (15–17) and from 16 to 44% for the advanced adenoma (5, 18, 19).

Many reasons could contribute to this phenomenon such as the composition of populations (20), ambient temperatures (21, 22), and manufacturers (23). One of the most important reasons might be the quality control of fecal sample collection. CRC and adenoma, especially the advanced adenoma, usually bleed slightly and intermittently. Blood from the colonic lesions would not distribute homogeneously on the surface of the feces. Non-standard and low-quality sample collections could leave out the hemorrhage, which would lead to a missed diagnosis. Many people, especially older adults or people who are participating in CRC screening for the first time, might be more likely to have the non-standard sample collection. Unqualified fecal sample collection could lead to a false-negative qFIT result (24), delay further diagnostic colonoscopy, and even cause death. To avoid the influence of low-quality sample collection, increasing sample collection operant training would contribute to the diagnostic efficiency of the qFIT in CRC screening.

Few studies have evaluated the influence of the sample collection quality on CRC screening. This pilot study aimed to preliminarily assess whether the practical fecal sample collection operant training could improve the diagnostic accuracy of the qFIT in CRC screening and to evaluate whether improving the quality of fecal sample collection could contribute to CRC screening.

## Materials and Methods

### Design

This study had a prospective, observational, and cross-sectional design. Ethical approval was granted by the Medical Ethics Committee of the Qilu Hospital of Shandong University [KYLL-2019(KS)-348]. The study was registered at ClinicalTrials.gov (No. NCT04454099). In this study, all the patients had never used the FIT previously and were invited to collect the two qFIT samples from the different bowel movements before colonoscopy. The patients did not receive the practical operant training for the fecal collection before the first qFIT collection. After collecting the first sample, the patients completed another qFIT sample. The collection of the first fecal sample was represented as the operant training for the second qFIT sample and the first and second qFIT samples represented those without the operant training and after operant training, respectively. After completing the two qFIT samples, patients underwent colonoscopy within a week. Patients who underwent colonoscopy and the two qFIT samples were enrolled in the final analysis. The primary outcome was the sensitivity and specificity of the qFIT without and after the operant training for CRC and advanced colorectal neoplasia (ACRN). The secondary outcome was the difference in the area under the curves (AUCs) for CRC and ACRN and the concentrations of the fecal Hb between the qFITs without and after the operant training.

### Patients

The study included the consecutive patients scheduled to undergo colonoscopy from the outpatient clinics and wards in the Qilu Hospital of Shandong University between July 2020 and March 2021. According to the Asia-Pacific Colorectal Screening (APCS) score (25), patients aged 50–75 years old from the moderate- or high-risk populations were enrolled. None of the patients had previously used the FIT. The possible APCS scores for each risk factor are as follows: 0: age <50 years, 2: 50–69 years, 3: >70 years; 1: male sex, 0: female sex; 2: family history of CRC in a first-degree relative, 0: no family history of CRC in a first-degree relative; and 0: nonsmoking, 1: smoking. Three risk stratifications were defined: average (0–1), moderate (2–3), and high risk (4–7). The exclusion criteria were as follows: (a) low risk by the APCS; (b) history of intestinal surgery; (c) history of CRC; (d) history of inflammatory bowel disease, ischemic enteritis, vascular malformation of the intestine, or other diseases that could result in the intestinal tract bleeding; (e) symptoms including visible rectal bleeding, hematuria, severe and acute diarrhea, and the Bristol feces score of 7 (26); (f) pregnancy, lactation, or menstrual phase; and (g) severe congestive heart failure or other severe diseases, causing the patients to not tolerate the complete colonoscopy. Demographic information was obtained from all the patients.

### Fecal Sample Collection and the Quantitative Fecal Immunochemical Tests

Patients received one-on-one fecal sample collection education and a leaflet (including sample collection steps, a two-dimensional code to obtain the demonstration video, and consulting telephone) from the investigators. They could learn actively through the information in the leaflet when they had trouble during the process of collection of the fecal sample. Next, they received a qFIT kit, including a qFIT tube and a plastic box. Patients defecated the feces into the box and scraped the surface of each segment by using a tailored sample probe that could quantitatively collect 2 mg of the feces. Patients inserted the sampling probe into the collection tube and ensured that the feces were dissolved in 2 ml Hb-stabilizing buffer. After completing the fecal collection, the patients submitted the first qFIT tube to the investigators. Then, the patients received another qFIT kit and completed the second qFIT tube. No dietary or medication restrictions were advised during the study. The fecal samples were stored at 4°C and submitted to the investigator within 1 day. Patients underwent colonoscopy within a week after the fecal samples were submitted.

After receiving the fecal samples, the investigator stored the tubes at 4°C. The samples were tested for 24 h. The investigator tested the qFITs by using the AC-SCREEN hs-qFIT analysis system (FUNOTEC Corporation Ltd., China) with a measurement range of ≥10 ng Hb/ml buffer solution. Fecal Hb was reported in μg/g (μg Hb/g feces) (27). According to the design of the qFIT kit, 10 ng Hb/ml buffer solutions equal 10 μg/g. Results lower than 10 μg/g were expressed as “1.0.” Each patient had the two qFIT results including the qFIT without the operant training and after the operant training. The qFIT detection was blinded to the information and colonoscopy results of the patients. The qFIT results were independently sent to a statistician.

### Colonoscopy and Histology

Colonoscopy is the acknowledged golden standard for colorectal disease and we chose colonoscopy as the reference standard. Patients who had a standard bowel preparation and complete colonoscopy were enrolled in the analysis. Standard bowel preparation was defined as a Boston Bowel Preparation Scale score (28) ≥2 for all the segments. Complete colonoscopy was defined as reaching the cecum or the lumen that was blocked because of the malignant lesions. Colonoscopies were performed by experienced endoscopists who performed more than 2,000 colonoscopy procedures. During the colonoscopy, the polyps were biopsied or removed and the CRCs were biopsied. According to the most advanced finding, the participants were classified as CRC, advanced adenoma, non-advanced adenoma, hyperplastic polyps, other colonic lesions, or normal. The proximal colon included the colon from the cecum to the splenic flexure and the distal colon included the descending colon to the rectum. Histological features included tubular, tubulovillous, villous, and serrated. Dysplasia was classified as either low or high grade. The size was estimated by using the calibrated open biopsy forceps, which were 6 mm in diameter. Advanced adenoma refers to the adenomas with a diameter of ≥10 mm, tubulovillous or villous adenomas, or high-grade dysplasia, regardless of the size. All the endoscopists and pathologists were blinded to the qFIT results.

### Statistical Analysis

At the beginning of this study, we calculated the sample size based on a significance level of 0.05 and power of 0.8 with the PASS version 15.0 (NCSS Statistical Software, Kaysville, Utah, USA). Based on the lowest acceptable sensitivity in the previous studies (0.80) and the prevalence of CRC in the Qilu Hospital of Shandong University (0.025), we calculated that a total sample size of 800 patients was required. Considering that the patients may have failed the sample collection, could not complete colonoscopy, or may cancel their colonoscopy, we estimated that a total of 1,000 patients would be required.

For this study, we analyzed the sensitivity, specificity, positive predictive value (PPV), and negative predictive value (NPV) for the qFITs in the different cutoffs from 10 to 100 μg/g, and all of them were calculated and reported with 95% CIs. The differences between the diagnostic accuracies of the qFIT without and after the practical operant training were tested by using the paired chi-squared test (29). Receiver operating characteristic (ROC) curves were plotted. Differences between AUCs were tested by using the DeLong test. Optimal sensitivity and specificity were obtained from the Youden index. The Wilcoxon signed-rank test was used to analyze the difference in the Hb concentrations between the qFIT without and after the operant training. Statistical significance was set at *p* < 0.05. All the analyses were performed by using the SPSS Statistics (version 24; IBM Corporation, Armonk, New York, USA), the MedCalc (version 19.6.1; MedCalc, Ostend, Belgium, UK), or the GraphPad Prism (version 7.04; GraphPad Software, San Diego, California, USA).

## Results

### Patients and Colonoscopy

**Figure 1:**
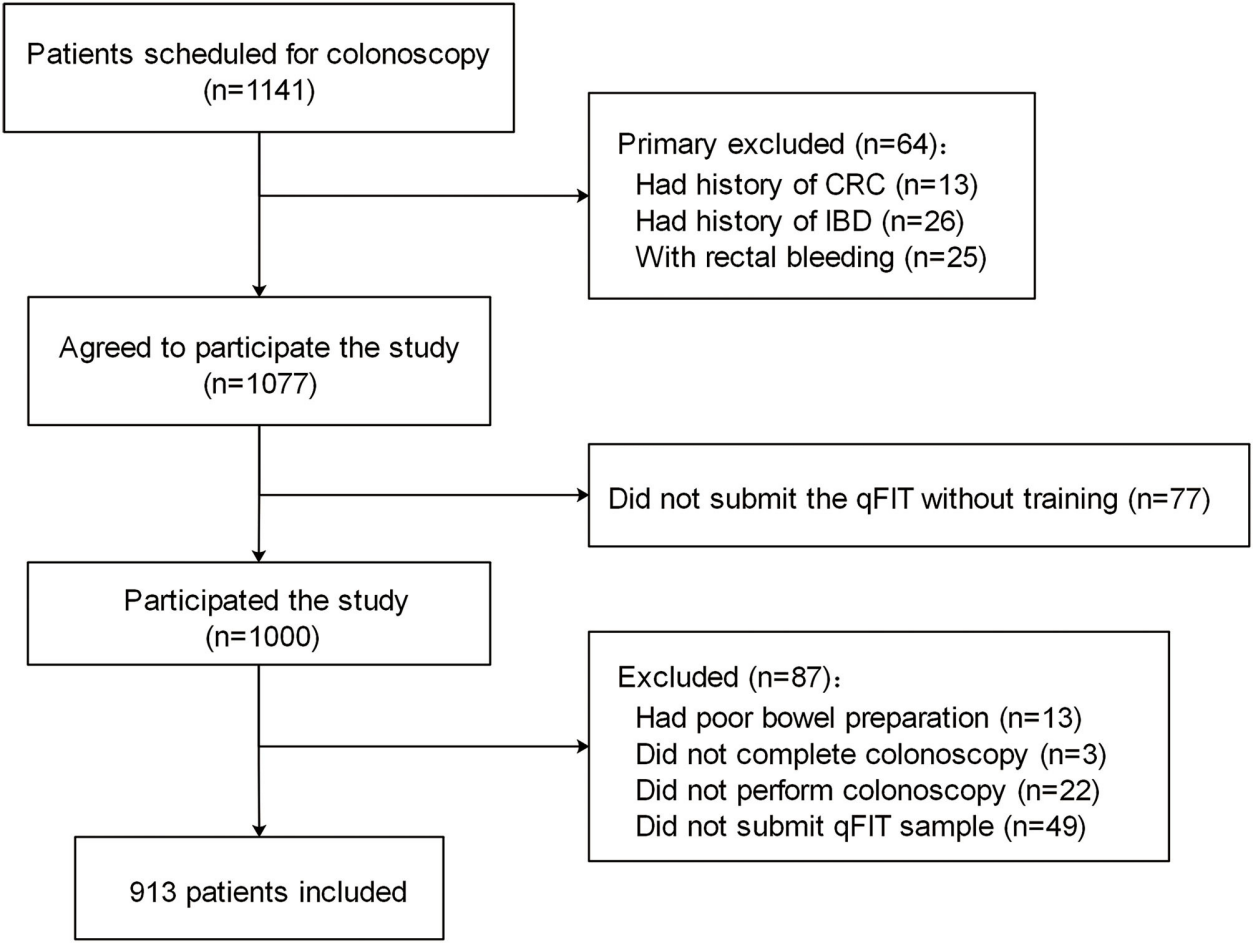
Study of the flow diagram. CRC, colorectal cancer; IBD, inflammatory bowel disease.

Figure 1 shows a flow diagram of the study. Out of the 1,173 patients scheduled for screening colonoscopy, 1,000 patients met the inclusion criteria and completed the first fecal sample collection. About 87 patients were excluded from the study. Finally, 913 patients who returned the qFIT samples and underwent the complete colonoscopy were included in the study analysis. No unexpected adverse events were observed during the study period. Demographic characteristics are summarized in Table 1. The patients included 51.3% men with a median age of 59.0 years [interquartile range (IQR) 54.0–64.5]. The main reason for colonoscopy was routine physical examinations (48.7%), including 407 (91.4%) for screening and 38 (8.5%) for surveillance.

**Table 1:** Population demographics.

**Demographics**	**Value**
Male, *n* (%)	469 (51.3)
Median age (IQR), year	59.0 (54.0 – 64.5)
Mean BMI (SD), kg/m^2^	24.2 (4.0)
**APCS**, ***n*** **(%)**
Moderate risk	669 (73.3)
High risk	244 (26.7)
**Reasons for colonoscopy**, ***n*** **(%)**
Physical examination	445 (48.7)
Abdominal pain, abdominal discomfort	296 (32.4)
Change in bowel habits	61 (6.7)
Diarrhea	46 (5.0)
Constipation	40 (4.4)
Weight loss	14 (1.5)
Anal symptoms	11 (1.2)
Family history of colorectal cancer in first-degree relatives,	40 (4.4)
*n* (%)	
Diabetes, *n* (%)	86 (9.4)
Hypertension, *n* (%)	219 (24.0)
Alcohol use, *n* (%)	228 (25.0)
Smoking, *n* (%)	213 (23.3)

*IQR, interquartile range; SD, standard deviation; APCS, Asia-Pacific Colorectal Screening Score*.

Overall, CRC was detected in 25 (2.7%) patients, including 4 (16.0%) patients in the proximal colon and 21 (84.0%) patients in the distal colon. Advanced adenoma was detected in 56 patients (6.1%). There were 273 (29.9%), 188 (20.6%), 92 (10.1%), and 279 (30.6%) patients with nonadvanced adenoma, hyperplastic polyps, other colonic lesions, and no colonic disease, respectively.

### Performance Characteristics of qFIT Without and After the Practical Sample Collection Operant Training for CRC and ACRN

The proportions of the patients positive at the thresholds of 10, 30, 50, and 100 μg/g of the qFIT without the operant training were 11.9, 9.0, 6.4, and 4.5%, respectively and 14.0, 10.1, 6.8, and 5.1% after the operant training, respectively. In the range from 10–100 μg/g, the sensitivities of the qFIT without and after the operant training for CRC ranged from 84.0–72.0 and 100.0–92.0%, respectively, and the specificities of the qFIT without and after the operant training for CRC ranged from 90.1–97.4 and 88.4–97.3%, respectively (Table 2). At the same cutoffs, the sensitivities of the qFIT without and after the operant training for ACRN ranged from 49.4–32.1 and 69.1–39.5%, respectively, and the specificities of the qFIT without and after the operant training for ACRN ranged from 91.7–98.2 and 91.3–98.2%, respectively. The specificities and NPVs of the qFIT after the operant training were similar, but the sensitivities and PPVs were higher compared to those of qFIT without the operant training at the same threshold. Similar results were observed for ACRN.

**Table 2:** Performance characteristics of the quantitative fecal immunochemical test (qFIT) without and after the practical sample collection operant training for colorectal cancer (CRC) and advanced colorectal neoplasia (ACRN).

**qFIT (μg/g)**		**Sensitivity (%)**	*P-*value*	**Specificity (%)**	***P-*value+**	**PPV (%)**	**NPV (%)**	**TP**	**FN**	**FP**	**TN**
**Colorectal cancer**										
10	Without	84.0 (63.1–94.7)		90.1 (87.9–91.9)		19.3 (12.6–28.2)	99.5 (98.6–99.8)	21	4	88	800
	After	100.0 (83.4–100.0)	0.134	88.4 (86.1–90.4)	0.151	19.5 (13.3–27.7)	100.0 (99.4–100.0)	25	0	103	785
30	Without	72.0 (50.4–87.1)		94.0 (92.2–95.5)		25.4 (16.1–37.3)	99.2 (98.2–99.6)	20	5	62	826
	After	96.0 (77.7–99.8)	0.041	93.8 (92.0–95.3)	0.883	30.4 (20.8–41.9)	99.9 (99.2–100.0)	25	0	67	821
50	Without	72.0 (50.4–87.1)		95.5 (93.9–96.7)		31.0 (19.9–44.7)	99.2 (98.2–99.6)	18	7	40	848
	After	96.0 (77.7–99.8)	0.041	95.7 (94.1–96.9)	0.866	38.7 (26.9–52.0)	100.0 (99.2–100.0)	24	1	38	850
100	Without	72.0 (50.4–87.1)		97.4 (96.1–98.3)		43.9 (28.8–60.1)	99.2 (98.3–99.6)	18	7	23	865
	After	92.0 (72.5–98.6)	0.074	97.3 (95.9–98.2)	1.000	48.9 (34.3–63.7)	99.8 (99.1–100.0)	23	2	24	864
**Advanced colorectal neoplasia**										
10	Without	49.4 (38.2–60.6)		91.7 (89.6–93.4)		36.7 (27.8–46.5)	94.9 (93.1–96.3)	40	41	69	763
	After	69.1 (57.8–78.7)	0.005	91.3 (89.2–93.1)	0.812	43.8 (35.1–52.8)	96.8 (95.3–97.9)	56	25	72	760
30	Without	38.3 (27.9–49.8)		95.2 (93.5–96.5)		43.7 (32.1–55.9)	94.1 (92.2–95.5)	34	47	48	784
	After	50.6 (39.4–61.8)	0.016	95.4 (93.7–96.7)	0.871	51.9 (40.4–63.2)	95.2 (93.5–96.5)	45	36	47	785
50	Without	35.8 (25.7–47.3)		96.5 (95.0–97.6)		50.0 (36.7–63.3)	93.9 (92.0–95.4)	29	52	29	803
	After	44.4 (33.5–55.9)	0.096	96.9 (95.4–97.9)	0.677	58.1 (44.9–70.3)	94.7 (92.9–96.1)	36	45	26	806
100	Without	32.1 (22.4–43.5)		98.2 (97.0–98.9)		63.4 (46.9–77.4)	93.7 (91.8–95.2)	26	55	15	817
	After	39.5 (29.0–51.0)	0.114	98.2 (97.0–98.9)	1.000	68.1 (52.7–80.5)	94.3 (92.5–95.7)	32	49	15	817

*95 CIs within the brackets*.

p-value: difference of sensitivity (*) and specificity (+) between the qFIT without and after the operant training.

*PPV, positive predictive value; NPV, negative predictive value; TP, true positives; FN, false negatives; FP, false positives; TN, true negatives*.

### Diagnostic Efficiency of the qFIT Without and After the Practical Sample Collection Operant Training for CRC and ACRN

**Figure 2:**
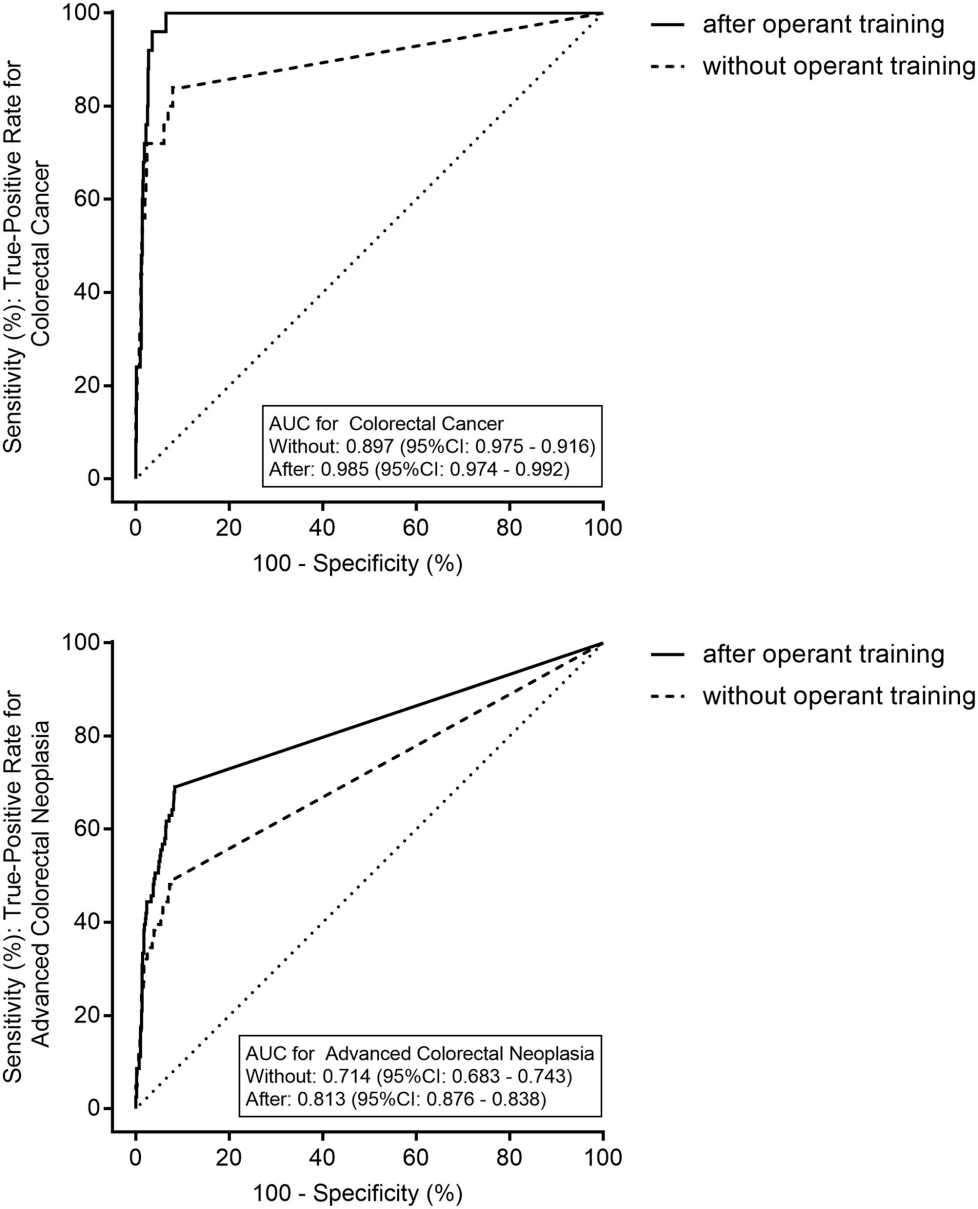
The receiver operating characteristics curves of the quantitative fecal immunochemical test (qFIT) without and after the practical sample collection operant training for colorectal cancer (CRC) and advanced colorectal neoplasia. AUC, the area under the curve.

Figure 2 shows the ROC curves of the qFIT without and after the operant training for CRC and ACRN. The AUCs of the qFIT without and after the operant training for CRC were 0.897 (95% CI 0.875–0.916) and 0.985 (95% CI 0.974–0.992), respectively, and those of ACRN was 0.714 (95% CI 0.683–0.743) and 0.813 (95% CI 0.786–0.838), respectively. The AUC of the qFIT after the operant training was significantly higher compared to qFIT without the operant training for CRC (*p* = 0.027) and ACRN (*p* = 0.001). The optimal sensitivity and specificity of the qFIT before the operant training were 84.0% (95% CI 63.1–94.7%) and 92.1% (95% CI 90.1–93.8%) for CRC (at 15 μg/g) and 49.4% (95% CI 38.2–60.6%) and 91.7% (95% CI 89.6–93.4%) for ACRN (at 10 μg/g). The optimal sensitivity and specificity of the qFIT after the operant training were 100.0% (95% CI 83.4–100.0%) and 93.6% (95% CI 91.7–95.1%), respectively, for CRC (at 28 μg/g) and 69.1% (95% CI 57.8–78.7%) and 91.3% (95% CI 89.2–93.1%), respectively, for ACRN (at 10 μg/g).

### Distribution of the Concentration of the Hb of the qFIT Without and After the Practical Sample Collection Operant Training

**Figure 3:**
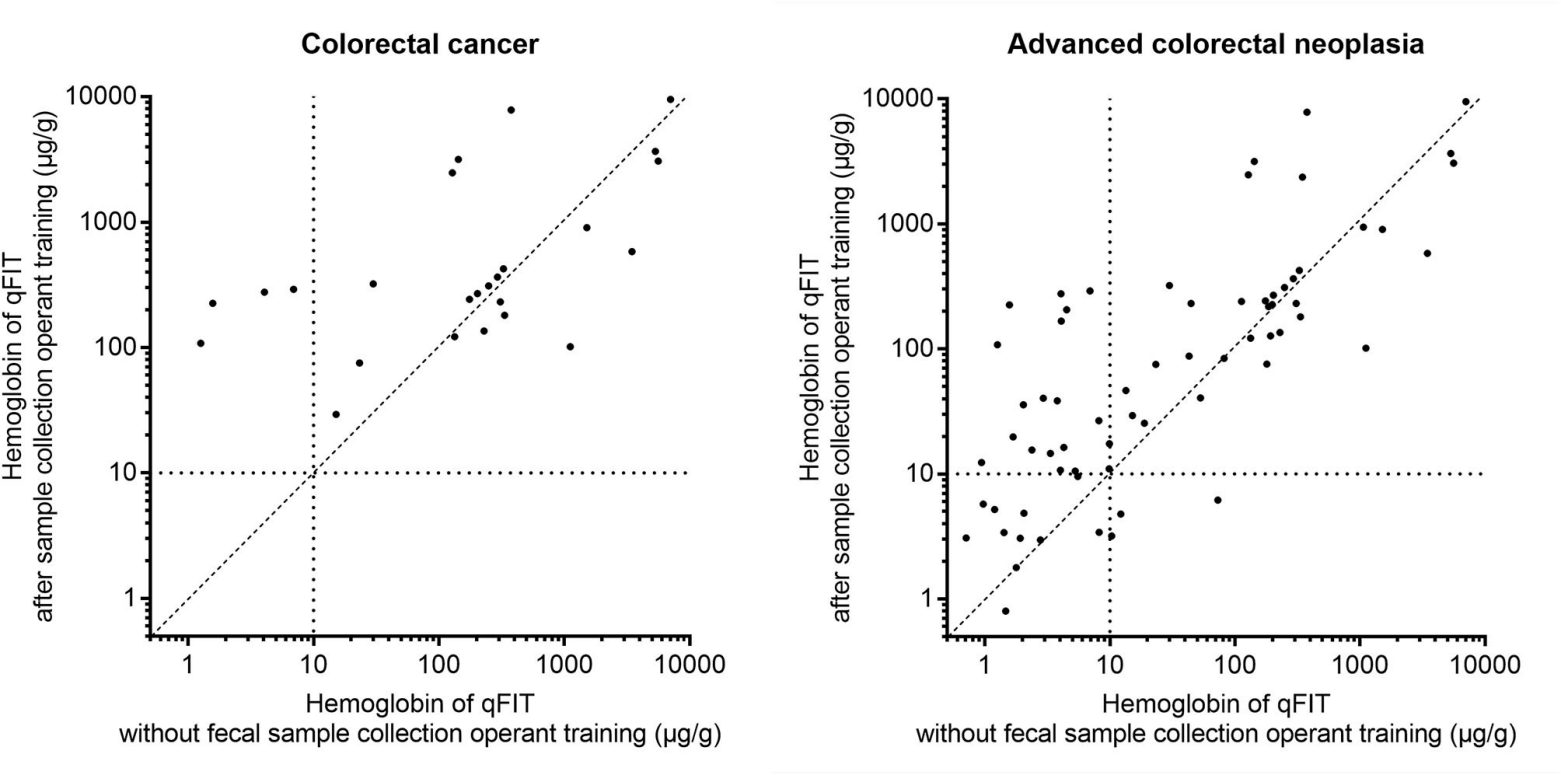
The distribution of the concentration of the hemoglobin (Hb) for the qFIT without and after the practical sample collection operant training for CRC and advanced colorectal neoplasia (ACRN). Each point represents the measurements of the Hb of the qFIT for one patient. The horizontal and vertical dotted lines show the limit of quantitation of the instrument. The oblique line divides the points into two parts. Points above/under the oblique line show the individuals whose detection value of the qFIT after the practical operant training was higher/lower than that without the operant training. Points in the upper left quadrant indicate the concentration of the fecal Hb that was detected by the qFIT after the operant training but not detected by the qFIT without the operant training. Points in the right lower quadrant indicate the concentration of the fecal Hb that was not detected by the qFIT after the operant training but detected by the qFIT without the operant training.

The distribution of the concentrations of the Hb is shown in Table 3. The concentration of Hb of the qFIT after the operant training was significantly higher than that without the operant training for ACRN (*p* = 0.009), advanced adenoma (*p* = 0.010), and adenoma (*p* = 0.010). For CRC, the concentration of the Hb of the qFIT after the operant training was significantly higher than that without the operant training, but the difference was not significant (*p* = 0.367). Figure 3 shows that the practical operant training could improve the detection for CRC and ACRN by increasing the detection of the low concentrations of the fecal Hb (upper left quadrant).

**Table 3:** The distribution of the concentration of the hemoglobin (Hb) of the qFIT without and after the practical sample collection operant training.

	**Patients, *n* (%)**	**Fecal hemoglobin level, median (IQR)**, **μg/g**		
		**Without training**	**After training**	*P*-value^*^
Total	913 (100.0)	1.0 (1.0–1.0)	1.0 (1.0–1.0)	0.001
Colorectal cancer	25 (2.7)	229.0 (26.6–745.5)	291.9 (157.9–1690.5)	0.367
Adenoma	329 (36.0)	1.0 (1.0–1.0)	1.0 (1.0–1.0)	0.010
Advanced adenoma	56 (6.1)	1.0 (1.0–23.7)	11.4 (1.0–40.5)	0.010
All non-advanced adenoma	273 (29.9)	1.0 (1.0–1.0)	1.0 (1.0–1.0)	0.214
Advanced colorectal neoplasia	81 (8.9)	1.0 (1.0–177.3)	35.8 (1.0–231.5)	0.009
Hyperplastic polyp	188 (20.6)	1.0 (1.0–1.0)	1.0 (1.0–1.0)	0.723
Other colonic lesion	92 (10.1)	1.0 (1.0–1.0)	1.0 (1.0–1.0)	0.601
Normal	279 (30.6)	1.0 (1.0–1.0)	1.0 (1.0–1.0)	0.240

*Results <10 μg/g were expressed as “1.0”*.

* *p-value: In relation to the concentration of the fecal hemoglobin without the operant training*.

*IQR, interquartile range*.

## Discussion

This study found that performing the practical sample collection operant training could increase the diagnostic efficiency of the qFIT for CRC and ACRN mainly by improving the detection of the low concentrations of the fecal Hb. Quality improvement of the fecal sample collection could increase the diagnostic accuracy of the qFIT in CRC screening.

Although performed consistently in CRC screening, the qFIT has heterogeneity in the performance characteristics. The composition (20) and degree of risk (30) of the populations, the ambient temperature of sample storage (31), the manufacture of FIT (32), and the other factors could influence the performance characteristics. However, according to the previous studies, when using the same brand of the qFIT and the same Hb threshold in an average-risk population, the sensitivities still had the wide ranges of variation for CRC (75–100%) (15, 16) and advanced adenoma (16–44%) (5, 18, 33, 34). In the different studies, the optimal cutoff values and diagnostic accuracies were different (20, 35, 36). When considering all the influencing factors, the quality control of the sample collection by the patients would have a great influence and would be difficult to control. Hemorrhage of the colorectal lesions has an uneven distribution on the fecal surface. Poor quality of the sample collection could lead to the missed detection of the fecal Hb. It is essential to improve the quality of the sample collection through training. To the best of our knowledge, few studies have demonstrated the benefit of the quality control of the qFIT sample collection in CRC screening.

Theoretically, the diagnostic efficiencies of the qFITs in the different bowel movements in a short period were similar in CRC screening. However, in this study, the sensitivities and PPVs of the qFITs after the operant training were higher than those without the operant training for CRC and ACRN at all the cutoffs. The AUC of the qFIT after the operant training was significantly higher compared to the qFIT without the operant training for CRC (*p* = 0.027) and ACRN (*p* = 0.001). In a short space of time, under nearly the same ambient temperature, storage time, and detection method, the improvement of the diagnostic accuracy for CRC and ACRN was largely attributed to the fecal collection operant training. In a usual CRC screening, it might be difficult for the doctors to offer more education in fecal sample collection to improve the quality of screening. This study showed that the operant training from the patients could improve the quality of the sample collection and increase the diagnostic efficiency for CRC and ACRN.

Practical fecal sample collection operant training increased the diagnostic accuracy for CRC and ACRN mainly by improving the detection of minimal bleeding from colorectal neoplasms. The positivity rate of the qFIT after the operant training was higher compared to qFIT without the operant training. For ACRN and advanced adenoma, the concentration of the Hb after the operant training was significantly higher than that without the operant training (*p* = 0.009, *p* = 0.010). The sensitivities at 10 and 30 μg/g were significantly higher after the operant training for ACRN. Although there was no significant difference, the concentration of the Hb after the operant training for CRC was still higher than that without the operant training (*p* = 0.367). The sensitivities for CRC at 30 and 50 μg/g were significantly higher after the operant training. This indicated that the improved diagnostic efficiency of the sample collection operant training was mainly due to increasing the detection of the lower concentration hemorrhage of ACRN.

In addition, although the positivity rate of the qFIT was increased, the practical fecal sample collection operant training did not increase the false-positive rates (equal to 1-specificity) for CRC or ACRN obviously. The sensitivities and PPVs improved after the operant training, but the specificities and NPVs of the qFIT after the operant training were similar to those without the operant training at all the cutoffs for CRC and ACRN. The data in this study indicated that the increased Hb concentrations by the sample collection operant training were from CRC and ACRN, but not from the other patients. Sample collection operant training could effectively improve the diagnostic efficiency of CRC and ACRN.

The standard operating procedure for the sample collection involves several key steps. In general, the patients learn the collection steps of the qFIT through instruction and experience. However, we believe that despite having received standardized fecal sampling education, many people could still have trouble with the standard protocol, which further influences the diagnostic accuracy. Patients can become skilled through the repeated sample collection operation. Moreover, once the patients realize that they had difficulty with the first sample collection, they could actively seek guidance, which could also be helpful in being more proficient. This study showed that the sampling experience from the practical operant training could improve the effectiveness of the further qFIT sample collection and increase diagnostic accuracy. This phenomenon, sensitivities of qFIT after operant training were higher than those without operant training for CRC and ACRN, presents in all cut-off values. The sensitivities increased, but the specificities did not decrease, indicating the importance of the operant training.

The quantitative fecal immunochemical test has been widely used in population-based CRC screening and the diagnostic efficiency of the qFIT influences the CRC mortality and screening burden (37, 38). The diagnostic performance of the FIT is sometimes unsatisfactory. An increase in the sample test number may improve the detection rate and sensitivity for ACRN in CRC screening, but simultaneously might lead to the misdiagnosis and cause poor compliance. Park et al. (5) found that the two- (AUC, 0.914 vs. 0.887) or three-sample (AUC, 0.922 vs. 0.887) strategies provided the best discrimination compared with a one-sample strategy for cancer. Although the studies hypothesize that the two-sample FIT screening would be the most desirable strategy for diagnostic accuracy (35, 39, 40), many other studies reported that the diagnostic accuracy of the two tests is similar compared to one test (41, 42), especially for ACRN (5, 43). It could even decrease the completion of screening (44) and increase the cost of detecting CRC and ACRN (16). Increasing the diagnostic efficiency of a single test to reach multiple tests could be an appropriate approach. Therefore, it would be feasible to improve the diagnostic efficacy of a one-sample test by increasing the quality control of the sample collection. In this study, the diagnostic efficiency of the qFIT after the operant training for CRC was similar compared to the two-/three-sample qFIT, but the diagnostic efficiency of the qFIT without the operant training was similar compared to the one-sample qFIT. This suggests that the sample collection operant training before the formal collection could improve the detection rate for CRC and ACRN effectively; meanwhile, the single test after the operant training does not increase the medical cost and colonoscopy burden. This pilot study preliminarily verified our hypothesis and provided the basic data for a further large-sample randomized controlled trial.

In this study, the patients included were the asymptomatic average-risk individuals and the symptomatic patients. The qFIT would be more sensitive and specific because of the symptomatic patients. Levi et al. (35) had similar population characteristics and sample size to our study. The sensitivities and specificities for CRC at 10 and 30 μg/g in this study were similar to those of the three-sample tests in Levi's et al. (35). For ACRN, the sensitivity and specificity were also similar. The optimal thresholds for the two- and three-sample tests were 75 ng/ml (equal to 25 μg/g), which were close to that of the FIT after the operant training. In some studies of the asymptomatic average-risk populations, the appropriate thresholds were ~20 μg/g (16). However, a meta-analysis of the studies on the patients with the high-risk symptoms reported that the optimal fecal Hb threshold was between 10 and 20 μg/g. Therefore, the thresholds in the different populations must be chosen according to the different population characteristics.

This study has some limitations. First, this was a single-center study with a relatively small sample size. Enlarging the sample size might lead to significant differences in the concentration of the Hb for CRC between the qFITs without and after the operant training. Large-scale, multicenter clinical trials are needed to validate the possibility of this modified strategy. Second, some patients in this study underwent colonoscopy for the clinical indications and they were more likely to have a higher risk of ACRN compared to the general population. Further studies on the practical operant training in CRC screening are required for an asymptomatic screening population. Third, this was a cross-sectional study. Higher evidence quality requires further clinical randomized controlled trials.

In this study, we found that the diagnostic accuracy of the qFIT after the practical fecal sample collection operant training was superior to that without the operant training. The fecal sample collection operant training could improve the diagnostic accuracy for CRC and ACRN, mainly by improving the detection of the low concentration of bleeding. Quality improvement of the sample collection could contribute to the diagnostic efficiency of the qFIT in CRC screening.

## Data Availability Statement

The raw data supporting the conclusions of this article will be made available by the authors, without undue reservation.

## Ethics Statement

The studies involving human participants were reviewed and approved by the Medical Ethics Committee of Qilu Hospital of Shandong University. The patients/participants provided their written informed consent to participate in this study.

## Author Contributions

R-cZ participated in the conceptualization, data curation, formal analysis, investigation, methodology, supervision, and writing the original draft. P-zW participated in the data curation, formal analysis, methodology, writing the review, and editing. Y-yL and YZ participated in the conceptualization, investigation, writing the review, and editing. M-jM participated in the conceptualization, writing the review, and editing. F-yM participated in the data curation and investigation. CL participated in the investigation and methodology. X-yY and ML participated in the conceptualization and methodology. X-lZ participated in the conceptualization, methodology, and supervision. Y-qL participated in the conceptualization, funding acquisition, supervision, writing the review, and editing. All authors contributed to the article and approved the submitted version.

## Funding

This study was supported by the Clinical Research Center of Shandong University (Grant No. 2020SDUCRCA012) and the Innovation Team Project of Jinan (2019GXRC005) to Y-qL. This study was also supported by the National Natural Science Foundation of China (81873550 and 81670489) and the Taishan Scholars Program of Shandong Province to Y-qL.

## Conflict of Interest

The authors declare that the research was conducted in the absence of any commercial or financial relationships that could be construed as a potential conflict of interest.

## Publisher's Note

All claims expressed in this article are solely those of the authors and do not necessarily represent those of their affiliated organizations, or those of the publisher, the editors and the reviewers. Any product that may be evaluated in this article, or claim that may be made by its manufacturer, is not guaranteed or endorsed by the publisher.
